# GA-SMOTE-RF Enhanced Kalman Filter with Adaptive Noise Reduction

**DOI:** 10.3390/s26072165

**Published:** 2026-03-31

**Authors:** Yiming Wang, Hui Zou, Yuzhou Liu, Tianchang Qiao, Xinyuan Xu, Yihang Li, Changxun He, Shunv Zhou, Hanjie Wang, Qingqing Geng, Qiqi Song

**Affiliations:** Department of Electronic and Optical Engineering, Xianlin Campus, Nanjing University of Posts and Telecommunications, 9 Wenyuan Road, Qixia District, Nanjing 210023, China; huizou@njupt.edu.cn (H.Z.); b23022002@njupt.edu.cn (Y.L.); b23022112@njupt.edu.cn (T.Q.); b23041804@njupt.edu.cn (X.X.); b23020302@njupt.edu.cn (Y.L.); b23021816@njupt.edu.cn (C.H.); b24130301@njupt.edu.cn (S.Z.); b23021108@njupt.edu.cn (H.W.); b24020804@njupt.edu.cn (Q.G.); b24011502@njupt.edu.cn (Q.S.)

**Keywords:** genetic algorithm, SMOTE algorithm, random forest algorithm, Kalman filter, strategy classification

## Abstract

**Highlights:**

**What are the main findings?**

- The proposed intelligent system, integrating GA, SMOTE, RF, and Kalman filtering, achieves outstanding performance in both classification and signal recovery. It attains a high atmospheric turbulence interference classification accuracy of 98.27%, significantly outperforming traditional methods like SVM and KNN, while also demonstrating excellent adaptive noise reduction with an average RMSE of 0.6983 across 400 signal groups, zero estimated delay, and tightly controlled innovation sequence statistics (mean of −0.0049, standard deviation of 0.6960), ensuring high-fidelity signal recovery.
- Extensive validation on 283 real-world FSO measurement windows demonstrates superior adaptive noise reduction performance, characterized by an RMSE of 0.308 and an exceptionally low Average Regret of 0.75%, with consistent performance across different measurement days (21 December vs. 22 December), validating its robustness to temporal variations and confirming cross-day generalization capability.

**What are the implications of the main findings?**

- This study presents the first comprehensive validation of the GA–SMOTE–RF framework on real-world FSO data, bridging the longstanding gap between synthetic simulations and practical deployment scenarios, thereby providing a robust and real-time solution for mitigating atmospheric turbulence in free-space laser communication that can directly enhance the reliability and quality of communication in critical military and rescue operations.
- The successful integration of intelligent classification with adaptive filtering sets a new methodological benchmark, showing strong potential for extension to other challenging wireless communication scenarios affected by dynamic environmental interference.

**Abstract:**

Low-noise free-space laser communication has widespread applications in military and rescue fields, but atmospheric turbulence severely affects communication quality. This paper proposes an intelligent classification and adaptive noise reduction system that integrates genetic algorithms (GA), synthetic minority oversampling technique (SMOTE), random forest (RF), and Kalman filtering, significantly improving turbulence channel interference classification accuracy and communication quality. Simulation results show that the system achieves a classification accuracy of 98.27%, with corresponding F1-score of 0.9732 and MCC of 0.9653, far exceeding algorithms such as SVM and KNN. After noise reduction, the average RMSE for 400 signal groups is 0.6983, with zero estimated delay, and the mean and standard deviation of the innovative sequence are −0.0049 and 0.6960, respectively, demonstrating excellent signal quality and efficient real-time processing capabilities. Beyond synthetic simulations, we conducted real-world FSO data studies to validate practical applicability. A 24-h field experiment collected 283 real FSO measurement windows, on which the proposed GA–SMOTE–RF method achieves 0.308 RMSE and 0.75% Average Regret in Kalman filter parameter selection, outperforming KNN and SVM, confirming practical applicability for real-world FSO systems.

## 1. Introduction

Random forest (RF), as a representative ensemble learning algorithm, has been widely used in voting, classification [1,2,3], and regression tasks because of its high accuracy, strong stability, good anti-interference capability, broad applicability, and robust generalization performance [4,5]. These advantages make RF particularly attractive for signal processing and decision-making problems in complex communication environments. In free-space optical (FSO) or laser communication systems, especially underwater military missions, emergency rescue links, air-to-ground optical links, inter-UAV optical interconnects, and other high-dynamic deployment scenarios, the communication channel is frequently degraded by turbulence, beam wander, intensity fluctuation, and background interference. In such cases, the ability to rapidly extract channel features and recommend an appropriate denoising or filtering strategy is critical for maintaining communication quality.

The communication systems that may benefit from this study mainly include intensity-modulated/direct detection (IM/DD) laser communication systems, such as those employing OOK, PPM, PAM, and OFDM-based optical intensity transmission, as well as related short- and medium-range FSO links where the received signal is strongly affected by turbulence-induced intensity fading. In addition, the proposed framework can also be extended to coherent optical or structured-light communication systems, including PSK/QAM-based coherent links and orbital angular momentum (OAM) multiplexed optical links, provided that the corresponding received signal descriptors or channel features are available. Therefore, the present study is not limited to a single physical link type; rather, it provides a general machine-learning-based strategy selection framework for turbulence-impaired laser communication systems.

Early studies have already demonstrated the potential of RF and hybrid machine learning methods for classification-related signal processing tasks. In 2014, Saraswat et al. [6] proposed a binary random forest feature selection method, which improved the handling of high-dimensional data and achieved relatively accurate classification results at low computational cost. However, that method still suffered from variable-type bias, limited ability to capture feature interactions, low stability, and underestimation of the importance of highly correlated variables. In 2017, Araújo et al. [7] combined RF with VM and artificial neural networks (ANN) and used Gaussian-difference-based preprocessing to improve predictive accuracy and robustness. Nevertheless, the method introduced higher computational cost and a stronger dependence on large training datasets. In 2019, Balasubramanian and Ananthamoorthy [8] combined wavelet clustering with RF, which improved robustness and partially reduced computational burden, but the overall complexity remained relatively high, and the generalization ability was still limited.

More recently, machine learning has provided new possibilities for noise reduction, signal recognition, and link adaptation in laser communication. In 2022, Abd El-Mottaleb et al. [9] identified OAM two-code optical signals under different meteorological conditions using KNN/SVM algorithms, which improved classification efficiency and reduced training cost. However, the model performance deteriorated in adverse weather and showed limited robustness under severe conditions. In 2024, Liu et al. [10] proposed a CNN with channel attention mechanisms for identifying FSO modulation formats, improving both accuracy and robustness; however, the model complexity increased significantly, making it less suitable for applications with strict real-time requirements. Cai et al. [11] later used deep learning to recognize composite OAM vortex patterns under moderate turbulence, achieving high recognition accuracy and partial end-to-end automation. Despite these advantages, the method still faced problems such as high training and inference cost, focus on a fixed class of OAM patterns, and limited generalizability to broader link conditions.

These studies indicate that existing approaches in laser communication mainly fall into two categories: (i) signal/modulation identification methods, such as OAM pattern recognition and modulation-format classification, and (ii) link-quality-oriented adaptation methods, such as denoising, filtering, or parameter optimization. The present work belongs primarily to the second category. Specifically, our method is designed to predict the optimal denoising/filtering strategy from measured channel and signal features, rather than to directly classify modulation formats. However, the proposed framework is naturally compatible with modulation classification or identification tasks. If the training labels are redefined as modulation classes (e.g., OOK, PPM, PAM, OFDM subtypes, PSK/QAM classes, or OAM mode labels), and if modulation-sensitive features are added, the same GA–SMOTE–RF architecture can be retrained for modulation recognition, signal-type identification, or joint channel-state/modulation-state classification. Therefore, the present framework should be viewed as a general adaptive learning architecture for turbulence-impaired laser communication, with denoising strategy recommendation as the main use case in this paper.

Traditional algorithms for adaptive optical signal processing generally suffer from one or more limitations, including high training cost, low prediction accuracy, weak generalization ability, and excessive model complexity. Consequently, there is a strong need for an algorithmic system that can learn rapidly from realistic channel conditions in high-dynamic and strong-turbulence environments, classify the communication state accurately, recommend a suitable denoising scheme with moderate complexity, and output effective filter parameters for adaptive noise suppression.

**Figure 1:**
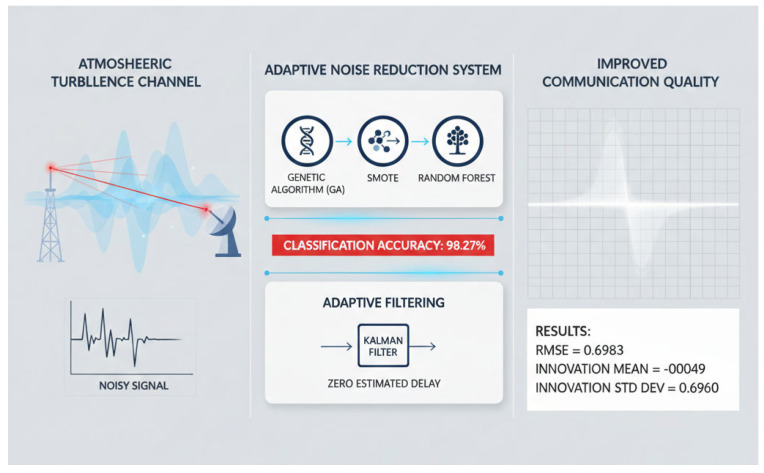
Conceptual diagram of article system overview.

To address this problem, we propose an intelligent strategy classification and adaptive noise reduction system integrating a genetic algorithm (GA), the Synthetic Minority Oversampling Technique (SMOTE), random forest (RF), and Kalman filtering, as shown in Figure 1. The framework fully exploits the advantages of RF, including its efficiency, fast inference, high classification accuracy, strong capability for handling multidimensional features, and good generalization performance [12,13,14]. By incorporating GA-based weight optimization, the proposed model further improves the global search ability of the strategy-label construction process [15,16,17], so that the resulting category labels better reflect actual communication performance. This improves the robustness and prediction accuracy of the downstream RF classifier. In the preprocessing stage, SMOTE is used to balance minority and majority classes [18,19], which preserves the feature space structure more effectively than conventional undersampling or naive repeated oversampling. As a result, the classifier becomes less biased toward dominant strategy classes and more reliable in distinguishing among different denoising strategies.

The trained RF classifier can then recommend the most suitable communication noise reduction parameter configuration under given channel conditions. Experimental results (Section 4) show that the proposed GA–SMOTE–RF–Kalman framework achieves stronger predictive accuracy, better generalization capability, and higher adaptability while maintaining moderate computational complexity. These characteristics make it a feasible and practical solution for adaptive noise reduction in turbulence-impaired free-space laser communication systems.

## 2. GA–SMOTE–RF System Modeling

**Figure 2:**
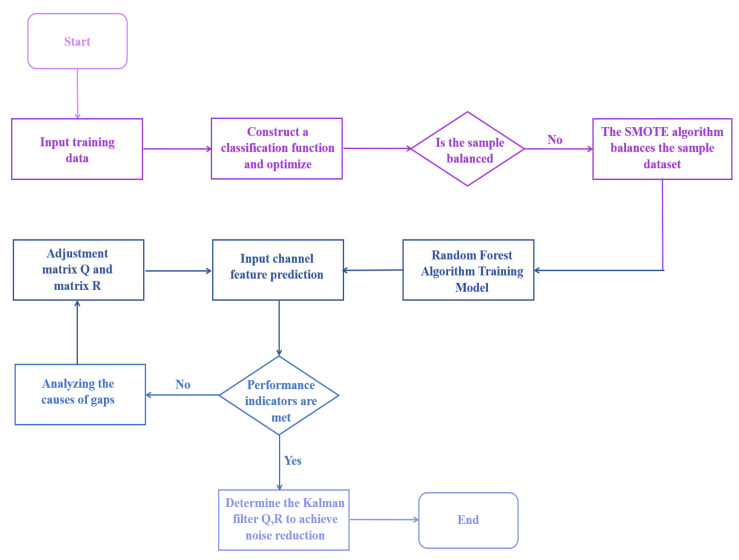
Flowchart of adaptive system design.

Figure 2 illustrates the conceptual framework of the proposed adaptive algorithm. The system achieves optimal noise reduction performance through dynamic adjustment of the Kalman filter’s process noise covariance matrix Q and measurement noise covariance matrix R. The channel characteristics in laser communication—including received light intensity mean ($P_{avg}$), spot drift velocity (${drift}_{speed}$), Fried parameter ($r_{0}$), turbulence structure constant ($C_{n}^{2}$), scattering entropy ($H_{entropy}$), and Zernike wavefront coefficients—exhibit a mapping relationship with the filter parameters Q and R. These parameters form a labeled set for multi-class classification:

(1) $$y=\left[\Delta f,L_{CP}\right]$$

The system considers a total of m distinct classification labels, allowing the label vector to be formally expressed as $Y=[y_{1},y_{2},\cdots ,y_{m}]$. The channel characteristics, comprising: (1) received light intensity mean ($P_{avg}$), (2) spot drift velocity (${drift}_{speed}$), (3) Fried parameter ($r_{0}$), (4) turbulence structure constant ($C_{n}^{2}$), (5) scattering entropy ($H_{entropy}$), and (6) Zernike wavefront coefficients($Z_{1},Z_{2},\;\cdots ,\;Z_{n}$), collectively form the channel feature vector:

(2) $$X=\left[x_{p_{avg}},x_{{drift}_{speed}},x_{Fried},x_{C_{n}^{2}},x_{H_{entropy}},x_{Z}\right]$$

Assuming that the channel features co-compose a feature vector $X\;=\;[x_{1},{\;x}_{2},\;\cdots ,{\;x}_{m}]$ of spatial size *m*, the mapping relationship between the channel feature vector and the parameter set of the Kalman filter of the adaptive system can be expressed as:

$$x_{i}\to y_{j}$$

(3) $$s.t.\{\begin{array}{c} x_{i}\epsilon X,1\leq i\leq n\\ y_{j}\epsilon Y,1\leq j\leq m \end{array}$$

The noise reduction performance is quantified through four key metrics:

(1) Root Mean Square Error (RMSE)

The root mean square error quantifies the average deviation between the filtered output and reference signal. A smaller RMSE value indicates higher estimation accuracy, defined as:

(4) $$RMSE=\sqrt{\frac{1}{N}\sum_{i=1}^{N}{\left(x_{filt}\left(i\right)-x_{true}\left(i\right)\right)}^{2}}$$

where $x_{filt}\left(i\right)$ denotes the Kalman output signal and $x_{true}\left(i\right)$ denotes the original input signal.

(2) Signal-to-Noise Ratio Gain (SNR Gain)

The SNR gain metric evaluates the filter’s effectiveness in enhancing the input signal’s signal-to-noise ratio, where higher values indicate superior noise reduction performance. The SNR Gain is calculated as:

(5) $${SNR}_{gain}={SNR}_{filt}-{SNR}_{raw}$$

where ${SNR}_{filt}$ represents the signal-to-noise ratio of the Kalman filter output and ${SNR}_{raw}$ denotes the signal-to-noise ratio of the raw input signal prior to noise reduction.

(3) Response Estimate Delay Time (Estimated Delay)

The response delay characterizes the filter’s tracking capability for abrupt signal variations, quantified in this study through maximum correlation hysteresis magnitude. The metric is formally defined as:

(6) $$\bar{s}=\frac{1}{N}\sum_{t=1}^{N}s\left(t\right),\bar{\hat{s}}=\frac{1}{N}\sum_{t=1}^{N}\hat{s}\left(t\right)$$

(7) $$R_{s,\hat{s}}\left(\tau \right)=\frac{\sum_{t=1}^{N}\left(s\left(t\right)-\bar{s}\right)\left(\hat{s}\left(t+\tau \right)-\bar{\hat{s}}\right)}{\sqrt{\sum_{t=1}^{N}{\left(s\left(t\right)-\bar{s}\right)}^{2}}\sqrt{\sum_{t=1}^{N}{\left(\hat{s}\left(t+\tau \right)-\bar{\hat{s}}\right)}^{2}}}$$

(8) $$\tau^{*}={argmax}_{\tau }\left\{R_{s,\hat{s}}\left(\tau \right)\right\}$$

(9) $$Estimated\;Delay=\left|\tau^{*}\right|$$

where $s\left(t\right),\;\hat{\;s}\left(t\right)$ represent the original and filtered output signals, respectively, $\bar{s}$ and $\bar{\hat{s}}$ denote the sample means of y(t) and ŷ(t), respectively, $R_{s\hat{,s}}\left(\tau \right)$ is the number of samples for normalized correlation computation, and Estimated Delay indicates the maximum correlation lag estimate.

(4) Innovation Sequence Residual Analysis

The innovation sequence residuals provide critical assessment of Kalman filter model matching, where ideal residuals should exhibit white noise characteristics. Filter performance is evaluated through three key residual properties: (1) zero-mean distribution, (2) stable variance, and (3) autocorrelation function (ACF) values approaching zero. The innovation sequence is formally defined as:

(10) $$e_{k}=z_{k}-H_{{\hat{x}}_{k|k-1}}$$

where $z_{k}$ represents the observed signal at time $k,{\;\hat{x}}_{k|k-1}$ denotes the a priori state estimate at time k given measurements up to k−1, and H is the observation matrix mapping state space to measurement space.

The Kalman filter parameter tuning problem can therefore be formulated as a constrained multi-objective optimization:

(11) $$\{\begin{array}{c} \min RMSE\\ \max{SNR}_{gain}\\ \min\left|\tau^{*}\right|\\ \min{\bar{e}}_{k}\\ \min Var\left(e_{k}\right) \end{array}$$

(12) $$s.t.\{\begin{array}{c} Q=Q^{T}\\ \forall v\;v^{T}Qv\geq 0\\ R=R^{T}\\ \forall v\;v^{T}Rv\geq 0 \end{array}$$

To overcome the computational complexity inherent in direct multi-objective optimization, this work develops an RF–GA-based classification framework that enables efficient adaptive parameter selection for laser communication noise reduction systems, significantly improving decision-making speed while maintaining performance.

## 3. Random Forest-Based Adaptive Algorithm

**Figure 3:**
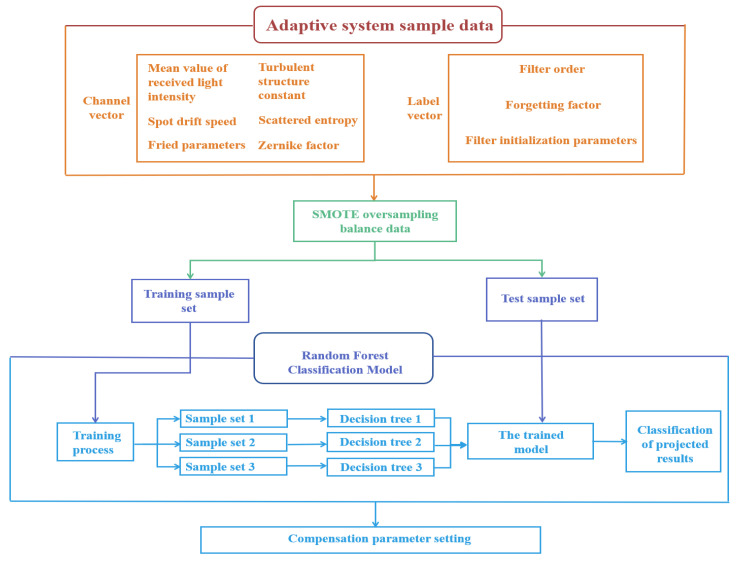
Flowchart of GA–SMOTE–RF algorithm.

Figure 3 illustrates the architecture of the proposed laser communication adaptive noise reduction system, which operates through six key processing stages:

Step 1: Feature Extraction

The feature extraction module obtains the channel feature vectors of the laser communication system, including the mean value of the received light intensity ($P_{avg}$), the spot drift velocity (${drift}_{speed}$), the Fried parameter ($r_{0}$), the turbulence structure constants ($C_{n}^{2}$), the scattering entropy ($H_{entropy}$), and the Zernike wavefront coefficients, and obtains real-time features for the subsequent system processing to provide a samples.

Step 2: Label Construction

The system evaluates samples through multiple feature vectors to generate a comprehensive score index:

(13) $$Score=W_{best}X^{T}$$

where

(14) $$W_{best}=\left[w_{best1},w_{best2},w_{best3},w_{best4},w_{best5},w_{best6},w_{best7}\right]$$

Thus, quantile-based mapping transforms channel features into label vectors.

Step 3: Genetic Algorithm Optimization

The GA iteratively optimizes the objective function (prediction accuracy) to converge toward optimal integrated score expressions.

Step 4: Sample Balancing

The SMOTE algorithm addresses class imbalance by oversampling minority categories to achieve balanced data distribution.

Step 5: Model Training

An enhanced random forest algorithm trains on balanced samples (with hold-out validation data), ultimately outputting strategy categories to guide filter parameter configuration.

Step 6: Real-Time Implementation

New channel features input to the system trigger real-time strategy recommendations, enabling dynamic optimization of Kalman filter matrices Q and R for optimal noise reduction.

### 3.1. Mapping Function Construction

The transformation from signal feature vectors to Kalman filter parameters Q and R necessitates rigorous classification criteria. Since different feature vectors variably influence signal filtering performance, we employ a linear mapping function (Equation (19)) to construct the empirical index. However, fixed-weight formulations suffer from three critical limitations: (1) subjective weight assignment, (2) lack of automated parameter optimization, and (3) inconsistent alignment with actual communication quality metrics.

To address these limitations, we implement a genetic algorithm (GA) that automatically optimizes the weighting coefficients through evolutionary computation. As illustrated in Algorithm 1, the GA iteratively refines the mapping function weights to ensure the derived strategy categories optimally represent the underlying communication performance characteristics. This approach eliminates subjective bias while maximizing the scientific validity of the classification labels.
**Algorithm 1.** Genetic Algorithm Optimization Procedure Step 1: Initialization Generate initial population, set weight vector initial values [0.15, 0.3, 0.5, 30, 0.3, 0.3] and account for feature magnitude differences. Step 2: Fitness Function Definition Carefully define the fitness function with the explicit objective of maximizing prediction accuracy, formulated as f(w)=−evalScoreAcc(w), where evalScoreAcc evaluates label matching accuracy after quartile-based discretization of weighted scores. Step 3: Parameter Configuration Set population size, define maximum generations, specify crossover probability, determine mutation probability and establish variable dimensions and constraints. Step 4: Evolutionary Process Perform selection, execute crossover, apply mutation, evaluate fitness and Update population while termination condition is not met. Step 5: Result Extraction Return optimal weight vector.

**Figure 4:**
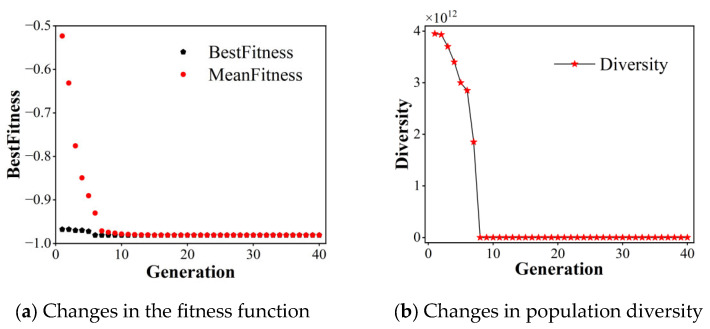
Graph of variation in performance of genetic algorithm.

Figure 4 illustrates the evolutionary dynamics of the genetic algorithm, demonstrating the convergence characteristics of fitness function values and population diversity throughout successive iterations.

Figure 4 presents the convergence characteristics of the genetic algorithm, showing the evolutionary trajectory of both fitness function values and population diversity across generations. The results demonstrate that as the evolutionary process progresses, the fitness function consistently converges toward −1 while population diversity exhibits a gradual decline, visually confirming the algorithm’s optimization capability through successive iterations.

### 3.2. Sample Preprocessing

To enhance the classification accuracy and robustness of the laser communication adaptive algorithm across diverse channel conditions, this study implements systematic preprocessing of raw sample data through two key operations: feature normalization and ZCA whitening. The methodological details of each preprocessing stage are elaborated as follows:

#### 3.2.1. Feature Normalization

Given the substantial magnitude disparities among laser communication channel feature vectors—where spot drift velocity typically ranges in the order of 0~10 m/s while turbulence structure constant ($C_{n}^{2}$) exhibits $10^{-15}\sim 10^{-13}$ scale variations—standard normalization is applied to each feature vector to ensure balanced contribution during model training:

(15) $$X_{norm}=\frac{X-u_{X}}{\sigma_{X}}$$

where $u_{X}$ and $\sigma_{X}$ represent the column-wise mean and standard deviation of features, respectively. This preprocessing step effectively eliminates scale-induced bias, enhances model convergence efficiency, and establishes uniform feature measurement scales by normalizing all inputs to zero mean and unit variance [10].

#### 3.2.2. Zero-Phase Component Analysis Whitening (ZCA Whitening)

To address potential covariance coupling among normalized features that may exhibit strong inter-feature correlations, we implement ZCA whitening to achieve approximate feature independence. The whitening transformation proceeds through three principal computational stages:

First, the empirical covariance matrix $cov(X_{nrom})$ is constructed from the normalized feature data. Subsequent eigenvalue decomposition of ${EDE}^{T}\;$ yields the orthogonal eigenvector matrix E and diagonal eigenvalue matrix diag(D). The complete whitening transformation is then computed as:

(16) $$\sum =cov\left(X_{norm}\right)$$

(17) $$\sum ={EDE}^{T}$$

(18) $$X_{ZCA}=X_{norm}Ediag\left(\frac{1}{\sqrt{diag\left(D\right)+\varepsilon }}\right)E^{T}$$

where ε represents a small numerical stabilization constant. This transformation effectively decorrelates the feature space while preserving the original data structure, enabling more accurate identification of individual feature contributions during model training.

### 3.3. Sample Balancing

In practical laser communication channels, the physical asymmetry and scarcity of poor-channel samples lead to severe class imbalance in strategy labels. For instance, among the 9 BER-based strategy categories, high-BER strategies (Strategies 8–9) typically comprise <10% of samples. This imbalance biases classifiers toward majority classes during training, degrading generalization through minority class neglect.

To address this, we employ the Synthetic Minority Oversampling Technique (SMOTE), which generates synthetic minority class samples via feature space interpolation. For each minority sample $x_{i}\epsilon R^{d}$, SMOTE:

Identifies its k-nearest neighbors ($x_{i1},\cdots ,x_{ik}$).

Synthesizes new samples:

(19) $$x_{new}=x_{i}+\lambda \left(x_{in}-x_{i}\right)$$

where λϵ(0, 1) controls interpolation and $x_{in}\epsilon (x_{i1},\cdots ,x_{ik})$. This constructs locally linear feature distributions while preserving inter-class continuity. The specific process is shown in Algorithm 2.
**Algorithm 2.** SMOTE Oversampling Procedure Step 1: Class Analysis Compute sample counts for each class and identify majority ($N_{max}$) and minority classes. Step 2: Minority Sample Generation For each minority class sample, identify k-nearest neighbors ($N_{k(x)}$) in feature space (*k* = 5), randomly select ${x_{nn}\epsilon N}_{k(x)}$ and generate synthetic sample: $x_{new}=x+\lambda (x_{nn}-x)$, where λϵU(0, 1). Step 3: Iterative Synthesis Repeat Step 2 until all minority classes reach target size $N_{max}$. Step 4: Dataset Reconstruction Construct balanced dataset.

The SMOTE algorithm effectively addresses class imbalance during training while simultaneously enhancing model robustness through strategic sample generation along decision boundaries. Crucially, its feature space linear interpolation preserves the underlying physical relationships governing real communication channels, ensuring synthetic samples maintain the statistical distributions and trends inherent to authentic channel conditions.

**Figure 5:**
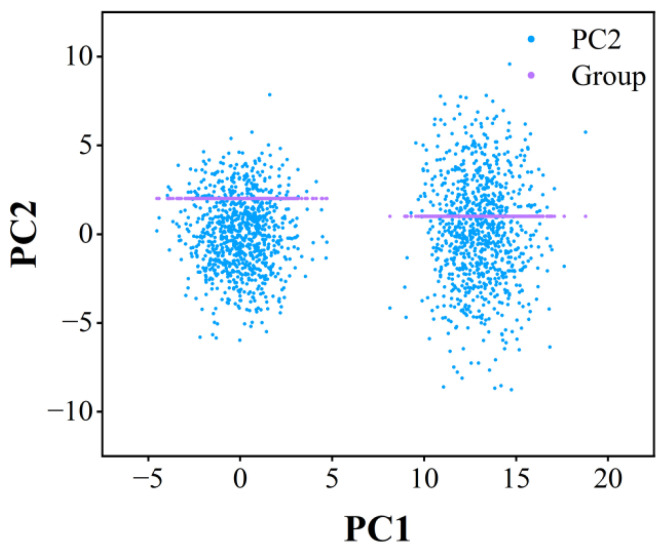
Comparison of data before and after processing by SMOTE algorithm.

The principal component analysis (PCA) projection shown in Figure 5 displays the different sample distributions before (blue) and after (purple) SMOTE processing. The post-SMOTE samples exhibit localized clustering in the feature space, confirming effective minority class augmentation. Subsequent histogram analysis will quantitatively validate the class distribution balancing achieved through this oversampling approach.

To properly evaluate classification performance under imbalanced conditions, accuracy alone can be misleading. For instance, if strategy categories 8–9 constitute only 8% of the dataset, then a trivial classifier predicting only the majority class would achieve 92% accuracy while failing completely to identify critical high-BER scenarios. Therefore, we employ a comprehensive set of evaluation metrics: precision (positive predictive value), recall (sensitivity), F1-score (harmonic mean of precision and recall), and the Matthews Correlation Coefficient (MCC). Among these, MCC is particularly informative as it produces a high score only when prediction performs well across all four confusion matrix categories, providing a balanced evaluation even with severely imbalanced class distributions. The specific algorithm performance results are shown in Section 4.

### 3.4. Adaptive Algorithm Flow

#### 3.4.1. Random Forest Mathematical Foundation

(1) Bootstrap Sampling with OOB Estimation

Given a training dataset of size n, the probability of a sample being excluded in a bootstrap sample is ${\left(1-\frac{1}{n}\right)}^{n}$. As n→∞, this probability converges to $e^{-1}\approx 0.368$, indicating approximately 36.8% of samples remain out-of-bag (OOB) per iteration.

(20) $$P_{Not\;selected}={\left(1-\frac{1}{n}\right)}^{n}\approx e^{-1}\approx 0.368$$

These OOB samples provide an unbiased estimate of the model’s generalization error, where lower OOB error corresponds to better generalization performance.

(2) Feature Randomization (Correlation Reduction)

Each decision tree employs a random subset of features for node splitting, ensuring diversity across the ensemble. This constraint simultaneously prevents dominant feature bias while encouraging exploration of alternative feature interactions, effectively reducing inter-tree correlation and lowering overall model variance through diversified voting mechanisms.

(3) Generalization Error Bound

The random forest generalization error (${PE}^{*}$) satisfies:

(21) $${PE}^{*}\leq \frac{\bar{\rho }\left(1-s^{2}\right)}{s^{2}}$$

where $\bar{\rho }$ represents average inter-tree correlation and s denotes individual tree strength. The error bound demonstrates that lower correlation between trees yields stronger ensemble generalization.

#### 3.4.2. Decision Tree Splitting Algorithm in Random Forest Classification

(1) Gini Impurity Metric

The Gini impurity quantifies the splitting quality in classification tasks by measuring the probability that two randomly selected samples possess divergent class labels. Defined as:

(22) $$Gini\left(t\right)=1-\sum_{k=1}^{K}{p_{k}}^{2}$$

where $p_{k}$ represents the proportion of class k samples at node t. Lower Gini values indicate higher node purity, with G(t)→0 denoting perfect classification.

(2) Splitting Criterion

The optimal split maximizes the Gini gain ΔGini:

(23) $$\Delta Gini=Gini\left(t\right)-\left(\frac{N_{left}}{N}Gini\left(t_{left}\right)+\frac{N_{right}}{N}Gini\left(t_{right}\right)\right)$$

where ΔGini quantifies the impurity reduction achieved by partitioning data on a specific feature. Higher ΔGini values correspond to more discriminative splits that significantly enhance classification performance. The complete splitting process is shown in Algorithm 3:
**Algorithm 3.** Random Forest Tree Splitting Step 1: Feature Evaluation For each randomly selected feature jϵ{1,⋯m}: Sort feature values and evaluate all possible split thresholds and compute Gini impurity reduction ΔGini for each candidate split. Step 2: Categorical Splitting For categorical features, evaluate all valid category grouping combinations. Step 3: Optimal Split Selection Identify and record the feature threshold pair (j,t) maximizing Δ*Gini*. Step 4: Termination Check If any condition is satisfied: Maximum tree depth reached ($d=d_{max}$) node sample count below minimum threshold (n $<n_{min}$) and perfect node purity (Gini=0). Then return as leaf node. Step 5: Recursive Splitting Partition data using (j,t) and repeat procedure for child nodes.

#### 3.4.3. GA–SMOTE–RF Adaptive Algorithm

The proposed adaptive framework integrates genetic algorithm (GA) for optimal mapping function weight optimization, synthetic minority oversampling technique (SMOTE) for class imbalance mitigation through feature space interpolation, and random forest (RF) for high-accuracy predictive modeling via ensemble learning, systematically addressing weight optimization, sample distribution balancing, and robust pattern recognition in a synergistic manner, with the complete algorithmic workflow illustrated in Algorithm 4 demonstrating this sequential integration for enhanced adaptive performance.
**Algorithm 4.** GA–SMOTE–RF Adaptive Algorithm Implementation Step 1: Data Preprocessing Process input data through feature normalization, GA-based weight optimization, and SMOTE oversampling to generate balanced training set ($X_{bal},Y_{bal}$). Step 2: Random Forest Configuration Initialize TreeBagger model with parameters: Numtrees = N(ensemble size), Maxnumsplits = M(maximum tree depth) and Numpredictorstosample = K(random feature subset size). Step 3: Ensemble Construction For each of N  trees: Generate bootstrap sample from ($X_{bal},Y_{bal}$), train decision tree using recursive splitting (Algorithm 3) and store trained tree in ensemble. Step 4: Prediction For test samples $X_{test}$: aggregate predictions from all N  trees and determine final classification $Y_{pred}$ via majority voting. Step 5: Performance Evaluation Compute standard metrics (accuracy, precision, recall) using ground truth labels.

The classification results are subsequently utilized to configure the Kalman filter parameters, enabling adaptive noise reduction optimized for the specific channel conditions identified by the classification output.

## 4. Simulation and Result Analysis

### 4.1. Sample Data Generation

Using MATLAB (2025B), we generated 900 distinct signal realizations and extracted their corresponding channel feature vectors, which were compiled into the feature matrix X. The genetic algorithm was then employed to optimize the weight coefficients for the label mapping function, with the derived optimal parameter configuration presented in Table 1.

The dataset exhibits inherent class imbalance, with minority categories (Strategies 8–9) comprising less than 10% of total samples—a realistic scenario reflecting the scarcity of severe turbulence conditions in practical laser communication channels. This imbalance necessitates careful evaluation beyond simple accuracy metrics.

Table 1 presents the optimized parameter combinations for the following channel characteristics: received light intensity mean ($P_{avg}$), spot drift velocity (${drift}_{speed}$), turbulence structure constant ($C_{n}^{2}$), scattering entropy ($H_{entropy}$), Fried parameter ($r_{0}$), Zernike second-order coefficients (wavefront tilt), and Zernike third-order coefficients (coma aberration).

The samples were partitioned into nine quantile-based categories using the mapping function, with each category assigned distinct label values according to its channel feature characteristics. Table 2 details the classification criteria and corresponding label assignments for each quantile division. The nine strategy categories (1–9) are derived from quantile-based division of the GA-optimized comprehensive score. Each category corresponds to a unique (Q, R) parameter pair for Kalman filter configuration, tailored to specific turbulence-induced channel conditions.

**Figure 6:**
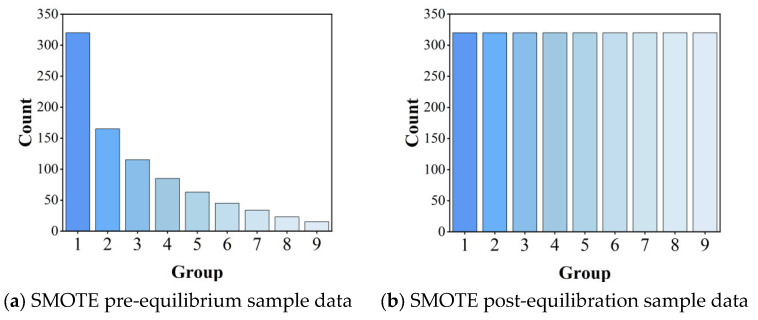
Histogram of sample comparison before and after processing by SMOTE algorithm.

To address the inherent class imbalance in the sample distribution, we employ the Synthetic Minority Oversampling Technique (SMOTE), which generates synthetic minority class samples through feature space interpolation. Figure 6 demonstrates the comparative sample distributions before and after SMOTE processing, illustrating the achieved class balance.

Figure 6 demonstrates the effectiveness of SMOTE in addressing class imbalance, showing significant improvement in sample distribution balance between the original and processed datasets. For model development, the dataset was partitioned into training (80%) and testing (20%) subsets, enabling robust evaluation of the random forest algorithm’s performance through holdout validation.

### 4.2. Sensitivity Analysis of the GA–SOMTE–RF Algorithm

**Figure 7:**
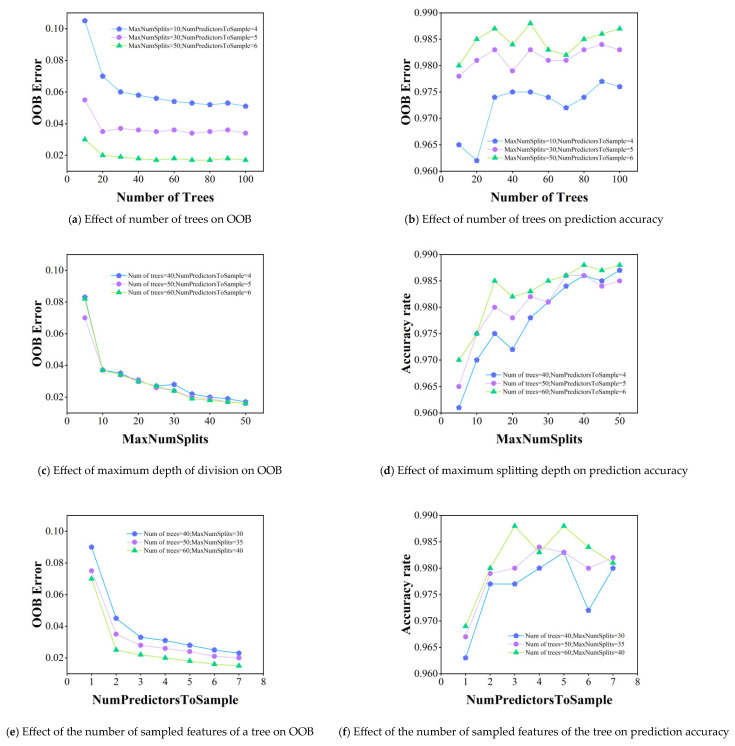
Effect of each parameter on model performance in random forests.

Three critical parameters governing random forest performance were systematically evaluated: (1) ensemble size (number of trees), (2) maximum tree depth, and (3) feature subset size per split. Using controlled experiments where two parameters remained fixed while varying the third, Figure 7 presents the corresponding out-of-bag (OOB) error and test set accuracy profiles, revealing the sensitivity of model performance to each parameter variation.

Figure 7 reveals three key observations for parameter selection: (1) the out-of-bag (OOB) error stabilizes when the ensemble size exceeds 50 trees, with optimal accuracy observed at 30–90 trees—a compromise between computational cost and performance led to selecting 50 trees; (2) both OOB error convergence and peak accuracy occur at a maximum tree depth of 40 splits; (3) feature sampling analysis shows stable OOB errors for ≥5 features and maximum accuracy at 3–5 features, justifying the selection of 5 features per split.

These optimized hyperparameters are summarized in Table 3.

### 4.3. Comparative Performance Evaluation

**Figure 8:**
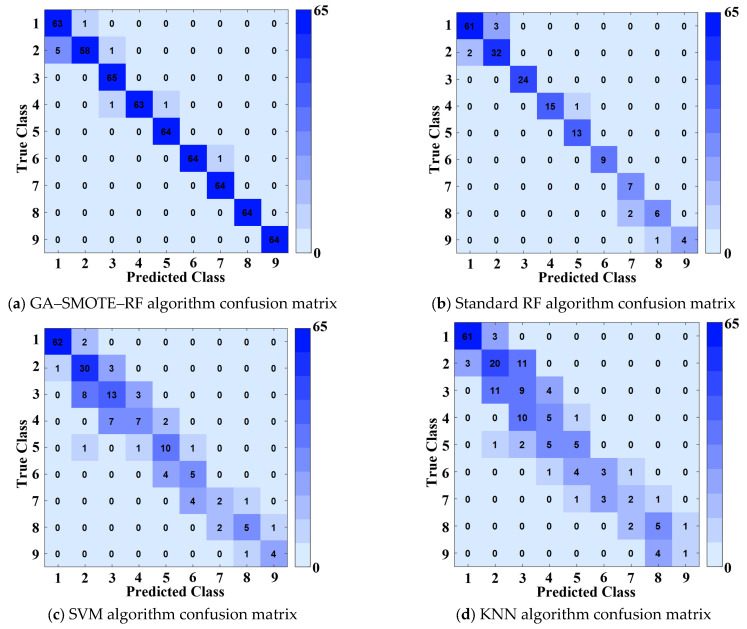
Comparison of confusion matrices for each algorithm.

To rigorously evaluate the GA–SMOTE–RF algorithm, we compare its prediction accuracy against three benchmark methods: (1) standard random forest, (2) support vector machine (SVM), and (3) k-nearest neighbors (KNN, k = 5). The comparative results, presented in Figure 8, demonstrate the superior classification performance of the proposed approach.

The nine strategy categories shown in the confusion matrices (Figure 8) correspond to the denoising strategies defined in Table 2. Specifically, Class 1 to Class 9 represent the nine distinct Kalman filter parameter configurations (Q, R pairs) optimized for different turbulence conditions, ranging from rapid response (Class 1) to strong process disturbance compensation (Class 9). These categories were established through quantile-based mapping of the GA-optimized comprehensive score index, ensuring each class corresponds to a unique combination of channel characteristics and filter parameters.

Figure 8 presents the confusion matrices comparing predicted versus actual classifications across all algorithms.

To rigorously evaluate classification performance under imbalanced data conditions, we report four complementary metrics beyond accuracy: precision, recall, F1-score, and the Matthews Correlation Coefficient (MCC). The MCC, in particular, provides a balanced measure even when classes are of very different sizes, as it considers all four confusion matrix categories (TP, TN, FP, FN).

As shown in Table 4, the proposed GA–SMOTE–RF algorithm achieves superior performance across all metrics: precision of 0.9770, recall of 0.9717, F1-score of 0.9732, and MCC of 0.9653. These results confirm that the high accuracy (98.27%) is not merely due to majority class dominance but reflects genuine classification capability across all nine strategy categories. In contrast, the KNN classifier’s relatively low MCC (0.4965) reveals its limited ability to distinguish minority classes despite its moderate accuracy (61.67%).

Table 5 further compares these results with state-of-the-art methods from the literature, confirming the competitive advantage of our approach.

As evidenced by Table 5, the proposed GA–SMOTE–RF algorithm achieves 98.27% classification accuracy despite the challenging conditions of multi-category classification, high-dimensional features, and complex interference patterns. These results demonstrate significant improvements in three key aspects: superior classification accuracy, enhanced generalization capability, and reduced computational complexity compared to existing approaches, confirming its strong practical applicability for industrial laser communication systems.

### 4.4. Noise Reduction Performance Evaluation

The proposed GA–SMOTE–RF adaptive system was rigorously evaluated using 400 randomly generated signal pairs (original and turbulence-corrupted). Quantitative analysis of the noise reduction performance was conducted by measuring five key metrics across all test cases: (1) root mean square error (RMSE), (2) signal-to-noise ratio (SNR), (3) estimation delay, (4) innovation sequence mean, and (5) innovation sequence standard deviation. The comprehensive results are presented in Table 6.

**Figure 9:**
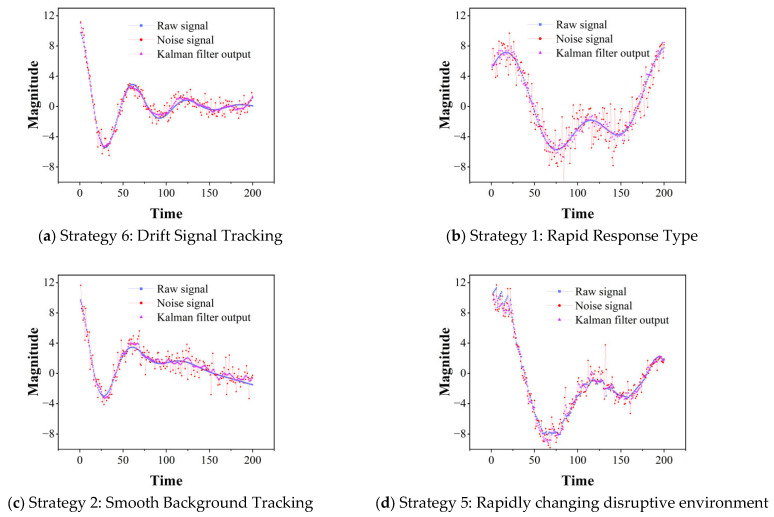
Visualization of the final noise reduction effect of the four groups of sample signals.

For qualitative evaluation, four representative signal pairs were selected from the test set to visually demonstrate the noise suppression capability of the proposed system. Figure 9 compares the original, corrupted, and filtered signals, providing intuitive validation of the algorithm’s effectiveness in recovering signal fidelity under turbulent conditions.

Figure 9 demonstrates the Kalman filter’s significant noise suppression capability, with the filtered signals closely approximating the original waveforms. Table 7 provides the corresponding quantitative metrics for these four representative cases, confirming the consistent noise reduction performance across different signal conditions.

Table 7 confirms significant signal quality improvement across all four representative cases, demonstrating the system’s robust noise reduction capability.

To comprehensively validate noise reduction performance, we analyze both overall statistics and per-class performance. Notably, the near-zero innovation sequence mean (−0.0049) and well-controlled standard deviation (0.6960) satisfy Kalman filter optimality conditions, confirming that the strategy classification accurately captures channel characteristics across all nine categories. Aggregate analysis of all 400 test signals reveals consistent performance: mean RMSE (0.6983), average SNR enhancement (3.1848 dB), near-zero estimation delay (0), and innovation sequence characteristics (mean: −0.0049; standard deviation: 0.6960), as detailed in Table 8.

As evidenced by Table 8, the system achieves exceptional noise reduction performance, characterized by: (1) minimal mean squared error (RMSE ≈ 0.6983), (2) significant SNR improvement (ΔSNR ≈ 3.1848 dB), (3) near-zero estimation delay, and (4) innovation sequence statistics (μ ≈ −0.0049, σ ≈ 0.6960) that satisfy the theoretical requirements for optimal Kalman filtering. These quantitative metrics collectively demonstrate the system’s superior noise suppression capability.

### 4.5. Real-World FSO Dataset Collection

To validate the practical applicability of the proposed framework, real-world FSO measurement data were collected from a 24-h continuous field experiment conducted on 21–22 December 2021. The experimental setup consisted of:

(1) Transmitter: 800 G coherent optical transmitter with PCS modulation
(2) Receiver: Coherent receiver with real-time signal acquisition capability
(3) Channel: 1.5 km free-space optical link subject to atmospheric turbulence
(4) Weather monitoring: IPMA weather station data (wind speed: 0–12 m/s, temperature: 5–18 °C, humidity: 45–85%)

The dataset comprises 283 valid time windows (144 from 21 December and 139 from 22 December), each containing 60 s of continuous signal acquisition. Extracted channel features include received optical power and intensity fluctuations, spot drift velocity derived from beam wander analysis, bit error rate (BER) and error vector magnitude (EVM) measurements, and signal-to-noise ratio (SNR) degradation.

This real-world dataset enables comprehensive evaluation of the proposed framework under practical turbulence conditions, addressing the limitation of synthetic-only validation in previous studies.

### 4.6. Experimental Results of FSO Dataset

Table 9 presents a comprehensive comparison of the proposed GA–SMOTE–RF algorithm against baseline methods (KNN, SVM, and Ridge regression) on the real-world FSO dataset.

The proposed GA–SMOTE–RF algorithm achieves superior overall performance across all key metrics. Notably, it attains the highest Exact Match Ratio of 23.74%, significantly outperforming KNN (15.11%) by 57% and surpassing SVM and Ridge (both 10.07%) by 136%. This indicates that the proposed method most frequently identifies the truly optimal Kalman filter configuration under real-world turbulence conditions.

More importantly, the proposed method achieves the lowest Average Regret of 0.75%, which is substantially better than KNN (1.09%) and far superior to SVM and Ridge (15.27%). Since Average Regret quantifies the performance loss when a non-optimal strategy is selected, the low regret of GA–SMOTE–RF implies that even in cases where the exact optimal strategy is not chosen, the selected strategy still yields near-optimal performance—a critical advantage for practical deployment where perfect matching cannot always be guaranteed.

In terms of prediction accuracy, GA–SMOTE–RF achieves an RMSE of 0.308 and MAE of 0.256, which are significantly lower than those of KNN (RMSE: 0.496, MAE: 0.442). The extremely high RMSE values of SVM (1.37 × 10^8^) and Ridge (2.47 × 10^9^) indicate severe prediction instability, making them unsuitable for practical applications. In contrast, the stable and accurate predictions of GA–SMOTE–RF ensure reliable decision-making.

Furthermore, the proposed method exhibits the lowest Median Regret of 0.61%, demonstrating consistent performance across the majority of test samples. Although its Max Regret (26.89%) is slightly higher than that of KNN (24.03%), the substantially lower Average and Median Regret confirm that GA–SMOTE–RF provides more stable and reliable strategy recommendations overall.

Collectively, these results demonstrate that the proposed GA–SMOTE–RF framework achieves superior strategy selection accuracy, lower performance loss, and more robust noise reduction compared to traditional methods, confirming its strong potential for practical FSO communication systems.

### 4.7. Limitations and Future Work

While the current simulation results provide strong proof-of-concept evidence for the GA–SMOTE–RF adaptive noise reduction framework, we recognize that experimental validation is essential to establish practical utility. The future work outlined above represents a systematic roadmap toward real-world deployment, with particular emphasis on hardware implementation, cross-environment generalization, and continuous parameter optimization.

## 5. Conclusions

This paper presents a novel GA–SMOTE–RF adaptive algorithm for laser communication systems, which dynamically optimizes Kalman filter parameters based on real-time channel characteristics. The proposed framework integrates three key innovations: a genetic algorithm (GA) for optimal mapping function weight optimization, synthetic minority oversampling (SMOTE) to address class imbalance, and a random forest (RF) classifier for accurate parameter prediction.

Experimental results demonstrate the algorithm’s effectiveness through:

(1) Superior classification performance with accuracy of 98.27%, F1-score of 0.9732, and MCC of 0.9653—the latter confirming robust minority class recognition.
(2) Robust noise reduction performance (RMSE  = 0.6983, ΔSNR = 3.1848 dB).
(3) Consistent performance across imbalanced class distributions, with precision and recall both exceeding 0.97, demonstrating genuine generalization capability.
(4) Superior generalization capability compared to existing approaches.
(5) Comprehensive real-data validation is conducted on 283 real FSO measurement windows from 24-h field experiments, demonstrating practical applicability beyond synthetic simulations. On this real-world data, the proposed method achieves superior strategy selection performance with an Average Regret of 0.75%, significantly outperforming KNN (1.09%) and SVM (15.27%).

The comparative analysis confirms the algorithm’s advantages in both computational efficiency and practical applicability for real-world laser communication systems under turbulent conditions.

## Figures and Tables

**Table 1:** Optimal parameter combinations for the system.

Parameters	Numerical Value
$w_{\mathrm{best}}$(1)	3.325
$w_{\mathrm{best}}$(2)	1.524
$w_{\mathrm{best}}$(3)	7.45 × 10^13^
$w_{\mathrm{best}}$(4)	0.939
$w_{\mathrm{best}}$(5)	27.66
$w_{\mathrm{best}}$(6)	7.102
$w_{\mathrm{best}}$(*7*)	1.296

**Table 2:** Policy mapping assignments.

Strategy	$\mathrm{Mapping}\;\mathrm{Function}\;\mathrm{Values}\;\times 10^{3}$	Typology	Q	R
1	0.2935~0.4421	Rapid response	0.050	0.20
2	0.4421~0.5906	Smooth background tracking	0.020	0.05
3	0.5906~0.7391	Low process noise	0.005	0.10
4	0.7391~0.8876	High-precision observation	0.010	0.01
5	0.8876~1.0362	Fast changing interference environment	0.100	0.20
6	1.0362~1.1847	Drift signal tracking	0.001	0.10
7	1.1847~1.3332	Medium turbulence compensation	0.010	0.10
8	1.3332~1.4817	High observation error	0.00	0.30
9	1.4817~1.6303	Strong process disturbances	0.20	0.10

**Table 3:** Adaptive system hyperparameter settings.

Hyperparameterization	Parameter Value
Number of decision trees	50
Maximum depth	40
Maximum number of features	5

**Table 4:** Comparison of algorithmic prediction performance.

Algorithm	Prediction Accuracy	Precision	Recall	F1-Score	MCC
GA–SMOTE–RF	98.27%	0.9770	0.9717	0.9732	0.9653
RF	95.00%	0.9735	0.9670	0.9689	0.9584
SVM	76.67%	0.6240	0.5389	0.5411	0.6674
KNN	61.67%	0.4676	0.4161	0.4309	0.4965

**Table 5:** Comparison of the performance of this paper’s algorithm and existing algorithms.

Arithmetic	Turbulence	Classification Accuracy	Model Complexity	Generalizability	Algorithmic Advantages of This Paper Explained
Modulation recognition CNN [10]	Strong	≈96.29%	High	medium	Neither accuracy nor applicability is as good as in this paper
Jamming environment LG pattern recognition [20]	Medium	≈92.00%	medium	medium	Low precision and task limitations
Composite vortex beam recognition [11]	Medium strong	≈93.23%	High	low	Low accuracy and poor generalization
FSO-OCDMA code recognition (KNN/SVM) [9]	Strong	≈97.00%	low	medium	Accuracy slips in bad weather, averages less than this article
GA–SMOTE–RF algorithm	Strong	≈98.27%	medium	High	High precision and light deployment

**Table 6:** Generated samples.

Sample Number	Recommended Strategy Category	RMSE	SNR Enhancement/dB	Estimated Delay/s	Sequence Mean	Sequential Standard Deviation
1	6	0.5680	6.06	0	0.0493	0.5673
2	7	0.7653	2.78	0	0.1238	0.7571
3	2	0.6846	3.14	0	0.0023	0.6863
⋯	⋯	⋯	⋯	⋯	⋯	⋯
399	1	0.5762	5.36	0	−0.0206	0.5773
400	5	0.6542	4.01	0	0.1570	0.6366

**Table 7:** Four groups of sample signal noise reduction effect data.

Recommended Strategies	6	1	2	5
RMSE	0.4907	0.5251	0.5334	0.4909
SNR enhancement/dB	5.1700	6.0300	5.0900	5.2900
Estimated delay/s	0.0000	0.0000	0.0000	0.0000
Sequence mean	0.0046	0.0083	−0.0359	0.0313
Sequential standard deviation	0.4919	0.5264	0.5336	0.4911

**Table 8:** Overall processing results of four hundred sets of sample signals.

Parameters	Numerical Value
RMSE	0.6983
SNR enhancement/dB	3.1848
Average estimated delay/s	0.0000
Average innovation series mean	−0.0049
Average innovation series standard deviation	0.6960

**Table 9:** Experimental Table of FSO Dataset.

Method	ExactMatch	Top2Match	Average Regret	Median Regert	MaxRegret	RMSE	MAE
KNN	0.1511	0.7122	0.0109	0.0135	0.2403	0.4964	0.4416
SVM	0.1007	0.0719	0.1527	0.1384	0.5042	1.3737 × 10^8^	1.3711 × 10^8^
Ridge	0.1007	0.0719	0.1527	0.1384	0.5042	2.4656 × 10^9^	2.4033 × 10^9^
GA–SMOTE–RF	0.2374	0.6907	0.0075	0.0061	0.2689	0.3082	0.2557

## Data Availability

The original contributions presented in this study are included in the article. Further inquiries can be directed to the corresponding author.
